# Critical Methodological Concerns in Electronic Nose Colorectal Cancer Detection

**DOI:** 10.14309/ctg.0000000000000961

**Published:** 2026-01-05

**Authors:** D.V. Kleemans, N.K. de Boer, S. Bosch

**Affiliations:** 1 Department of Gastroenterology and Hepatology, Amsterdam Gastroenterology Endocrinology Metabolism Research Institute, Amsterdam University Medical Centre, Vrije Universiteit Amsterdam, Amsterdam, The Netherlands.

With great interest, we read Wang et al's article on exhaled breath volatile organic compound (VOC) analysis for colorectal cancer (CRC) detection (1). Although the reported diagnostic accuracy appears promising, several methodological limitations warrant discussion and question the clinical applicability of these findings.

Sensor Drift: The absence of sensor drift correction protocols represents a methodological limitation. The Cyranose320 polymer-based sensors exhibit substantial baseline drift over short periods of time due to environmental factors, sensor aging, and varying background VOC exposure. Sensor response characteristics require careful consideration because drift can compromise eNose measurement reliability and diagnostic accuracy. We demonstrated that Cyranose320 sensor drift affects (fecal) VOC profiles more than clinical features within just 4 days of measurement, leading to significant differences in diagnostic accuracy when comparing data before and after thorough drift correction. In addition, the correction R/R0 = (Rmax−R0)/R0 was deemed insufficient (2). Reliable and generalizable test performance can therefore only be ensured after appropriate drift correction. The applied preprocessing steps, including ambient air correction and peak normalization, address certain aspects of data correction, but do not cover the aspects of sensor drift. Providing details on calibration frequency, environmental controls, correction algorithms, and the influence of measurement timing would be of value.

Study Design Limitations: Although a case-control design can aid initial feasibility assessment, it is prone to selection bias and may overestimate diagnostic performance in real-world contexts. Previous studies have shown that medication use (e.g., proton pump inhibitors), smoking, diet-driven microbial fermentation, and body mass index/cachexia significantly affect VOC profiles in urine, feces, and breath (3). These factors were incompletely recorded and unadjusted in the study by Wang et al. (1) Quite recently, Van Riswijk et al conducted a large multicenter prospective intention-to-diagnose study involving 3469 FIT-positive individuals undergoing endoscopy (4). Using the Aeonose for exhaled breath analysis, they failed to distinguish CRC (area under the curve [AUC] 0.542) and advanced adenoma (AUC 0.517). The larger sample size likely ensured a more balanced distribution of lifestyle factors influencing VOC composition, leading to a more realistic appraisal of clinical applicability.

Healthy controls: Unlike the CRC group, controls lacked endoscopic assessment to exclude colorectal disease. True validation requires endoscopically confirmed negative controls. Especially because advanced dysplasia or early CRC are often asymptomatic.

Bowel preparation: All patients with CRC underwent bowel preparation, colonoscopy, and biopsies before breath sampling, whereas healthy controls did not. The presumed short interval between diagnosis and breath sampling to avoid delaying CRC treatment may be relevant. Krishnamoorthy et al reported altered VOC profiles after bowel preparation, particularly increased acetone levels (5). This suggests bowel preparation can modify metabolic patterns, influence VOC profiles, and potentially introduce confounding factors.

The discordance between this study and van Riswijk et al's prospective validation in a large intention-to-diagnose cohort underscores why standardization in study design, sensor drift correction, endoscopically verified controls, and consideration of procedural confounders are essential to be able to sniff out the clinical potential of the eNose.

## CONFLICTS OF INTEREST

**Guarantor of the article:** S. Bosch, MD, PhD.

**Specific author contributions:** S.B.: accepting full responsibility for the integrity of the work and the decision to publish. D.V.K.: drafted the first version of the manuscript. S.B. and N.K.dB.: reviewed the manuscript and provided critical input.

**Financial support:** None to report.

**Potential competing interests:** D.V.K. and S.B. have nothing to declare. N.K.dB. has served as a speaker for AbbVie and MSD and has served as consultant and principal investigator for TEVA Pharma BV and Takeda. He has received a (unrestricted) research grant from Dr. Falk, TEVA Pharma BV, MLDS and Takeda. All outside the submitted work.
